# Association of neutrophil-to-lymphocyte ratio and risk of all-cause and cardiovascular mortality in adults with metabolic dysfunction-associated steatotic liver disease: a population-based cohort study

**DOI:** 10.3389/fmed.2024.1499524

**Published:** 2024-12-10

**Authors:** Ying Zhang, Ai-Hua Zhang, Rong-Li Li, Wen-Jun Li, Yun Liu, Teng Li

**Affiliations:** ^1^Department of Laboratory Medicine, Obstetrics and Gynecology Hospital of Fudan University, Shanghai, China; ^2^Department of Hematology, Weifang People's Hospital, Shandong Second Medical University, Weifang, Shandong, China; ^3^Department of Interventional Radiology, Weifang People's Hospital, Shandong Second Medical University, Weifang, Shandong, China

**Keywords:** metabolic dysfunction-associated steatotic liver disease, all-cause mortality, cardiovascular mortality, neutrophil-to-lymphocyte ratio, NHANES

## Abstract

**Background and objective:**

Inflammation is key to metabolic dysfunction-associated steatotic liver disease (MASLD) development. Nevertheless, the correlation between the inflammatory marker, neutrophil-to-lymphocyte ratio (NLR), and the MASLD prognosis remains unclear. We aim to determine the link between NLR and mortality risk in MASLD.

**Methods:**

The MASLD adult participants from the 1999–2018 National Health and Nutrition Examination Survey (NHANES) database were evaluated. Utilizing restricted cubic spline (RCS) analyses, as well as Cox proportional hazards (PH) models, the associations between NLR and all-cause mortality (ACM) and cardiovascular mortality (CVM) were analyzed in MASLD. Subgroup analyses and interaction tests were conducted to validate these associations. Moreover, we used sensitivity analyses to ascertain the robustness of the results.

**Results:**

Through 115 months of median follow-up, 2,307 of the 16,859 participants with MASLD died, including 650 deaths from cardiovascular causes. RCS analyses showed positive linear associations between NLR and both ACM and CVM. In the multivariable-adjusted Cox PH model, a one-unit elevation in NLR was related to a hazard ratio (HR) of 1.16 (95% confidence interval [CI]: 1.12–1.21) for ACM and 1.21 (95% CI: 1.15–1.27) for CVM. Participants were classified into higher (≥ 2.88) and lower (< 2.88) NLR groups employing the maximally selected rank statistics. The higher NLR group had a significantly elevated ACM (HR 1.38, 95% CI: 1.23–1.55) and CVM (HR 1.64, 95% CI: 1.32–2.03) risk compared to the lower NLR group. The associations were consistent in subgroup analyses based on age, gender, BMI, hypertension, and diabetes, with no significant interaction between NLR and these characteristics. Sensitivity analyses affirmed the main outcome’s robustness.

**Conclusion:**

A raised NLR independently predicts escalated ACM and CVM in MASLD.

## Introduction

Non-alcoholic fatty liver disease (NAFLD), a global predominant chronic liver disease, is defined by fat accumulation in liver cells. It is different from liver damage resulting from exaggerated alcohol consumption or other recognized factors. The disease spectrum comprises a continuum of conditions between simple steatosis and non-alcoholic steatohepatitis (NASH) and liver fibrosis, with further progression probability to cirrhosis or hepatocellular carcinoma (HCC) in certain individuals (1). The disease, which affects approximately 38% of the world’s population, has risen significantly since the 1990s and is emerging as a major public health concern (2–4). Recent developments in hepatology research have demonstrated that fatty liver disease correlates to other liver diseases and represents a systemic disorder correlated with metabolic dysregulation. In 2023, the multi-society Delphi consensus introduced “steatotic liver disease” (SLD) as an in-depth classification encompassing the diverse etiologies of hepatic steatosis. This updated system includes the reclassification of NAFLD as metabolic dysfunction-associated steatotic liver disease (MASLD) and NASH as metabolic dysfunction–associated steatohepatitis (MASH) (5). The MASLD definition emphasizes the significant cardiovascular and metabolic risk factors impact on the disease pathogenesis and progression (5). Studies have indicated similar clinical manifestations, histopathological findings, and disease prognosis between MASLD and NAFLD, suggesting that knowledge gained from studies of NAFLD over the past 30 years can be extrapolated to MASLD (6–8).

Inflammation is the main cause of NAFLD progression. Fat accumulation in the liver causes lipotoxicity, subsequently triggering an inflammatory response. The hepatic immune cell activation and the pro-inflammatory cytokine release recognize this response. These immune cells and cytokines exacerbate hepatocellular damage and facilitate the progression from simple steatosis to steatohepatitis (9). Furthermore, chronic inflammation induces the hepatic stellate cells (HSCs) activation, which is the principal mediator of liver fibrosis. Once activated, HSCs synthesize significant quantities of extracellular matrix proteins, thereby contributing to fibrosis, a pivotal indicator of NAFLD progression (10). Moreover, metabolic inflammation, a portion of which arises in the liver, is linked to several comorbidities, including cardiovascular disease and type 2 diabetes mellitus (T2DM), underscoring the systemic influence of NAFLD-correlated inflammation (11).

The neutrophil-to-lymphocyte ratio (NLR), a biomarker for various diseases, is calculated utilizing routine complete blood counts. The NLR indicates the balance between inflammation and immune responses and has recently garnered significant interest (12). The correlations between NLR and the risk and severity of liver disorders have been revealed, including NAFLD, liver cirrhosis, and HCC (13, 14). However, evidence regarding the prognostic value of NLR in MASLD adults remains limited. Identifying simple yet representative biomarkers for predicting survival in individuals with MASLD could significantly improve the clinical management of this condition. Therefore, the National Health and Nutrition Examination Survey (NHANES) data were employed to assess the associations between NLR and both all-cause mortality (ACM) and cardiovascular mortality (CVM) in the United States MASLD population.

## Materials and methods

### Data source and study population

Herein, we utilized the 1999–2018 NHANES data through the National Center for Health Statistics (NCHS). The NHANES survey was prepared to evaluate the health and nutritional status of the United States non-institutionalized civilian population. Approximately 10,000 individuals were selected biennially through complex sampling methods to participate in comprehensive assessments, including demographic data, physical examinations, biochemical and nutritional evaluations, and lifestyle questionnaires (15). Detailed documentation on NHANES can be accessed.^1^ The NCHS Institutional Review Board (IRB) authorized the original protocol, with all participants signing informed consent. Our study was exempted by our institution’s IRB and complied with the Strengthening the Reporting of Observational Studies in Epidemiology criteria (16).

**Figure 1:**
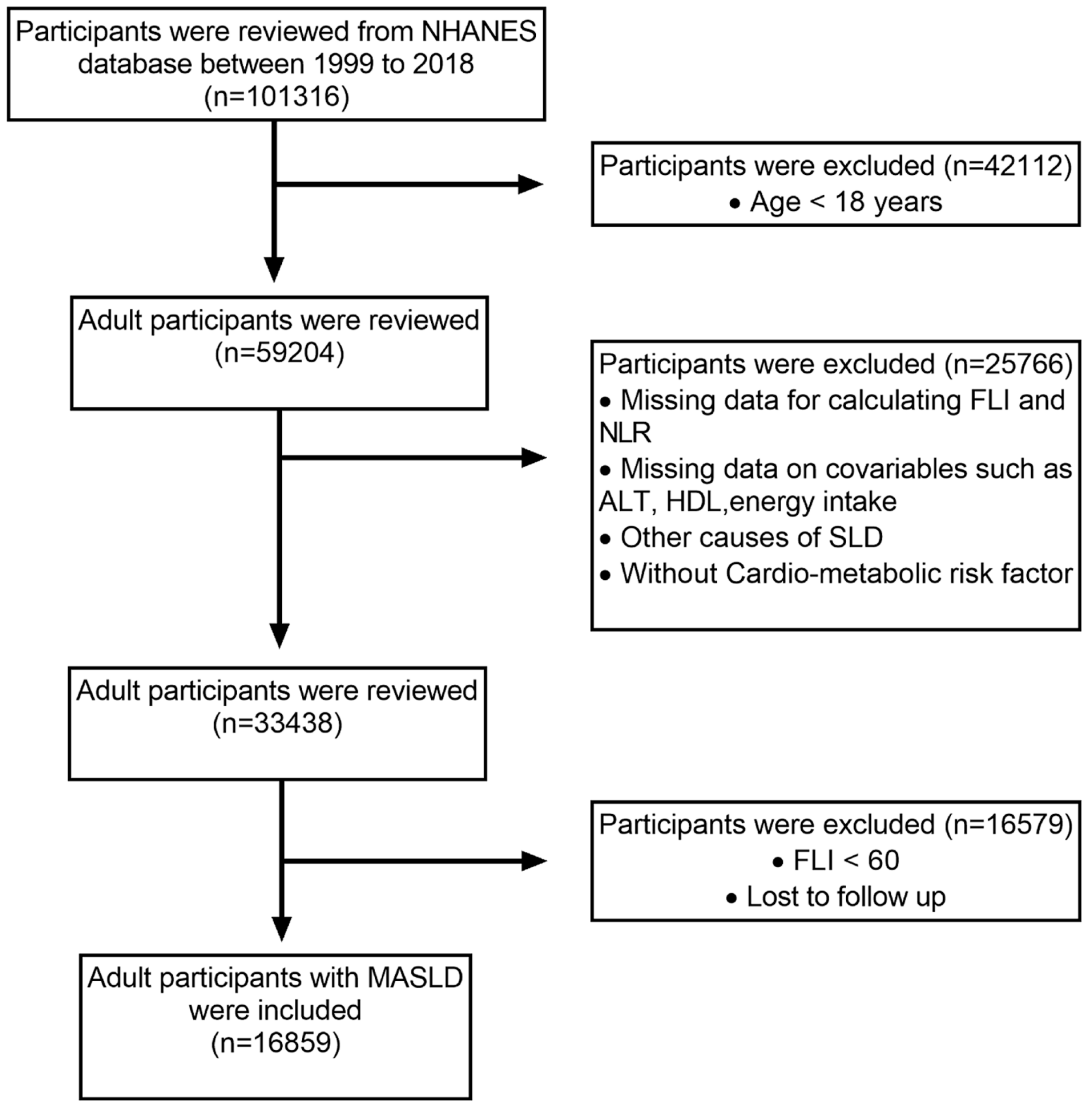
The participant selection process.

Out of the original 101,316 participants assessed over 10 interview cycles from 1999 to 2018, 59,204 individuals aged ≥18 years were considered eligible. Participants with incomplete data needed to calculate the fatty liver index (FLI), NLR, or related metabolic dysfunction factors were excluded. In addition, those with missing covariate data, such as alanine transaminase (ALT) levels and energy intake, were excluded from the analysis. Other exclusions included participants without SLD (FLI < 60), alternative causes of SLD, cancer diagnosis, and moderate to heavy alcohol consumption. After applying these criteria, 16,859 MASLD adult participants were included. Figure 1 illustrates the participant selection process.

### Assessment of MASLD

Since most interview cycles did not include an ultrasound assessment of hepatic steatosis, it was evaluated via the FLI, a reliable tool known for its high sensitivity and specificity. The FLI equation is as follows:


$$FLI=\frac{e^{0.953\times \ln\left(TG\right)+0.139\times BMI+0.718\times \ln\left(GGT\right)+0.053\times WC-15.745}}{1+e^{0.953\times \ln\left(TG\right)+0.139\times BMI+0.718\times \ln\left(GGT\right)+0.053\times WC-15.745}}\times 100$$

TG, BMI, GGT, and WC stand for triglyceride levels, body mass index, gamma-glutamyl transferase levels, and waist circumference, respectively. Based on previous studies, individuals with an FLI ≥ 60 were classified as having SLD (17, 18). The participants with viral hepatitis, autoimmune and alcohol-associated liver disease history, or daily alcohol consumption of >30 g for males and 20 g for females (daily alcohol consumption was evaluated using 24-h dietary recalls. For individuals who completed both recalls, the average alcohol intake derived from these two assessments was calculated. If only the first 24-h recall was completed, data from that single recall was used) were excluded to meet the Delphi diagnostic criteria for MASLD. Accordingly, MASLD was identified as FLI ≥ 60 accompanied by at least one cardio-metabolic risk factor presence, as described below:

1. BMI ≥ 25 kg/m^2^ (23 for Asia) or WC ≥ 94 cm for men and ≥ 80 cm for women or ethnicity adjusted equivalent;
2. Fasting blood glucose (FBG) ≥ 100 mg/dL or 2-h post-load glucose levels ≥140 mg/dL or hemoglobin A1c ≥ 5.7% or T2DM or therapy for T2DM;
3. Blood pressure ≥ 130/85 mmHg or receiving anti-hypertensive therapy;
4. Fasting plasma TG ≥ 150 mg/dL or receiving lipid-reducing medication;
5. Plasma high-density lipoprotein (HDL) < 40 mg/dL for men and < 50 mg/dL for women or those receiving lipid-reducing medication (5).

### Calculation of the NLR

The NLR for each participant was calculated from the parameters of complete blood count by the following equation:


$$NLR=\frac{\mathrm{Neutrophil counts}}{\mathrm{Lymphocyte counts}}$$

### Mortality outcomes

The utilized mortality data were provided by the NHANES public-use linked mortality files.^2^ Herein, the endpoints were deaths due to ACM and CVM. The International Classification of Diseases, Tenth Revision (ICD-10) categorized the cause-specific mortality. Specifically, CVM includes deaths from major cardiovascular and cerebrovascular diseases, as indicated by the following codes: I00–I09, I11, I13, I20–I51, and I60–I69. The follow-up period was measured from the baseline interview to either the mortality date or December 31, 2019 (19).

### Covariates

Depending on available literature and clinical evidence, the next covariates comprised age, gender, race/ethnicity, marital status, education level, household income, energy intake, BMI, diabetes, smoking status, hypertension, TG, WC, GGT, HDL, and ALT (19–21). Given that NLR is also correlated with liver fibrosis (22), which is a significant predictor of cardiovascular events and mortality in NAFLD (23, 24), we have additionally incorporated the fibrosis-4 (FIB-4) index. The FIB-4 index serves as a reliable non-invasive marker for assessing hepatic fibrosis in NAFLD (25). The FIB-4 equation is:


$$\mathrm{FIB}-4=\frac{\mathrm{Age}\times AST}{PLT\times \sqrt{\mathrm{ALT}}}$$

PLT stands for platelet counts and AST stands for aspartate transaminase. The NHANES survey assigned race/ethnicity into five groups: non-Hispanic White, non-Hispanic Black, Mexican American, other Hispanic, and other races. Education level was allocated into two groups: less than high school and more than or equal to high school. Marital status was allocated as either not married or married/living with a partner. Household income was determined depending on the poverty income ratio as low (< 1.30), moderate (1.30–3.50), and high (≥ 3.50) (26). Smoking status was divided into three groups: current (smoking >100 cigarettes during their lifespan and currently smoked), former (smoking >100 cigarettes during their lifespan but have stopped smoking), and never (smoking <100 cigarettes in their lifetime). Energy intake was evaluated by computing the mean of two values from two 24-h dietary recall interviews. BMI was assigned into under/normal weight (< 25 kg/m^2^), overweight (25–30 kg/m^2^), and obesity (≥ 30 kg/m^2^). The diabetes was characterized by FBG ≥ 126 mg/dL, hemoglobin A1c levels ≥6.5%, self-reported history of diabetes, and oral hypoglycemic agents or insulin utilization (27). Hypertension was characterized by systolic blood pressure ≥ 130 mmHg or diastolic blood pressure ≥ 80 mmHg, self-reported diagnosis of hypertension, or anti-hypertensive therapy utilization, depending on the 2017 American Heart Association/American College of Cardiology guidelines.

### Statistical analysis

Recognizing the complex and multi-faceted sampling strategy used by NHANES, which includes multistage, stratification, and clustering, we applied suitable sample weights to correct selection and non-response bias to affirm that the outcomes are representative of the United States general population.

Continuous variables showed deviations from a normal distribution, as indicated by the results of the Anderson-Darling test. Therefore, these variables were summarized using the median and interquartile range, while categorical variables were presented as numbers and weighted percentages. Herein, we employed the Wilcoxon Rank-Sum test for continuous variables and the Chi-Squared test for categorical variables to assess variances in baseline characteristics between groups.

In this study, we conducted restricted cubic spline (RCS) analyses to evaluate possible non-linear associations between NLR and both ACM and CVM. Knot points for the RCS curves were selected by minimizing the Akaike’s Information Criterion. The optimal NLR cut-off value was identified using maximally selected rank statistics. This cut-off value represented the most significant correlation to survival outcomes and was subsequently employed to classify individuals into higher and lower NLR groups. Survey-weighted Cox proportional hazards (PH) models were deployed to detect the links between NLR, ACM, and CVM in MASLD participants. Model 1 provided an unadjusted analysis, while Model 2 included adjustments for demographic variables comprising age, gender, race/ethnicity, marital status, and education level. The fully adjusted model (Model 3) further incorporated additional variables, including household income, smoking status, energy intake, BMI, WC, diabetes, hypertension, and serum levels of TG, GGT, HDL, ALT and FIB-4 index.

Additionally, Kaplan–Meier survival curves were deployed to clarify differential survival possibilities across NLR stratifications within the MASLD population, with statistical significance assessed using log-rank tests. Subgroup analyses were performed, considering variables including age, gender, BMI, diabetes, and hypertension, with an additional focus on examining their interactions. Furthermore, sensitivity analyses were employed to detect the primary outcome’s robustness, utilizing data from participants interviewed between 2003 and 2008, thereby addressing potential variations across different survey cycles.

The NLR predictive accuracy in forecasting survival within the MASLD population was evaluated by comparing the area under the curve (AUC) of time-dependent receiver operating characteristic (ROC) curves at various time points.

Imputation of missing values was deemed unnecessary, as participants with missing data had already been excluded during the initial screening process.

All statistical analyses were carried out at *p* < 0.05, which deemed statistical significance for a two-tailed *p*-value. Stata 17 (Stata Corporation, College Station, Texas, United States) and R 4.3.3 (R Core Team, Vienna, Austria) were used to analyze data.

## Results

### Baseline characteristics of participants

**Figure 2:**
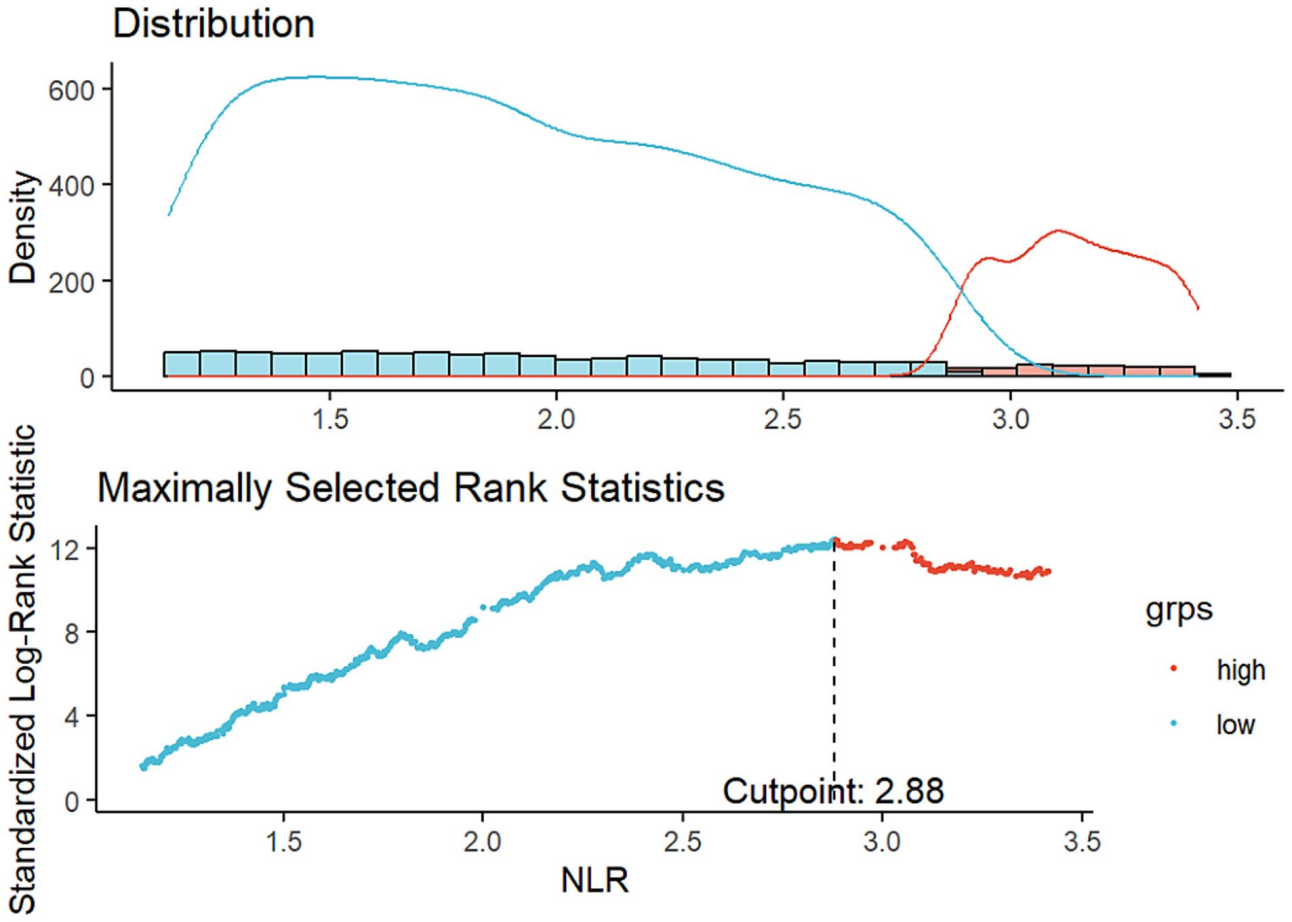
Assessment of the NLR cut-off value utilizing maximally selected rank statistics.

A cohort of 16,859 individuals across 10 survey cycles conducted between 1999 and 2018 were included. The participants had a median age of 47 years, with a higher proportion being males (8,614, 53.6%) than females (8,245, 46.4%). Most study populations were identified as non-Hispanic White (6,861, 65.4%). Approximately 20.4% of participants had an education level below high school, while 61.3% possessed a hypertension history, and 20.8% possessed a diabetes history. Additionally, 18.7% of the participants were current smokers during the survey. Throughout a median of 115 months of follow-up, 2,307 ACM and 650 CVM were recorded. Using an optimal NLR cut-off value of 2.88, which aligns with the most significant link to survival dependent on maximally selected rank statistics (Figure 2), participants were divided into two subgroups: those with a higher NLR (≥ 2.88, *n* = 3,108) and those with a lower NLR (< 2.88, *n* = 13,751). Analysis of baseline characteristics showed significant differences in age, WC, ALT, GGT, TG, HDL, FIB-4 index, race/ethnicity, marital status, smoking status, diabetes, and hypertension between individuals with higher and lower NLR. However, energy intake, gender, education level, BMI category, and household income were similar across both groups. Table 1 illustrates the participant’s baseline characteristics.

**Table 1:** Baseline characteristics of participants.

Variable	NLR group			*p*-value^2^
	Overall^1^ weighted *N* = 70,905,889 Unweighted *n* = 16,859	Lower NLR (<2.88)^1^ weighted *N* = 57,939,714 unweighted *n* = 13,751	Higher NLR (≥2.88)^1^ weighted *N* = 12,966,175 unweighted *n* = 3,108	
Age (years)	47 (35, 59)	47 (35, 59)	51 (37, 63)	<0.001
WC (cm)	110 (103, 119)	109 (103, 118)	112 (105, 121)	<0.001
ALT (U/L)	25 (18, 34)	25 (19, 34)	23 (18, 31)	<0.001
GGT (U/L)	25 (18, 38)	25 (18, 38)	24 (17, 38)	0.021
TG (mg/dL)	168 (118, 247)	169 (119, 248)	164 (113, 240)	0.013
HDL (mg/dL)	44 (37, 52)	44 (37, 52)	44 (37, 53)	0.032
Energy intake (kcal/day)	1,991 (1,505, 2,572)	1,990 (1,504, 2,573)	2,003 (1,517, 2,567)	0.783
FLI	86 (74, 95)	86 (74, 95)	88 (75, 96)	<0.001
FIB-4	0.84 (0.58, 1.23)	0.83 (0.57, 1.20)	0.88 (0.58, 1.35)	<0.001
NLR	2.00 (1.55, 2.63)	1.84 (1.46, 2.24)	3.44 (3.13, 4.12)	<0.001
Gender, %				0.392
Male	8,614 (53.6%)	7,061 (53.4%)	1,553 (54.7%)	
Female	8,245 (46.4%)	6,690 (46.6%)	1,555 (45.3%)	
Race/ethnicity, %				<0.001
MexicanAmerican	3,897 (11.1%)	3,256 (11.5%)	641 (9.0%)	
Other Hispanic	1,505 (6.0%)	1,241 (6.1%)	264 (5.6%)	
Non-Hispanic White	6,861 (65.4%)	5,228 (63.6%)	1,633 (73.5%)	
Non-Hispanic Black	3,561 (11.8%)	3,168 (13.0%)	393 (6.4%)	
Other Race	1,035 (5.7%)	858 (5.8%)	177 (5.6%)	
Education level, %				0.362
< high school	5,443 (20.4%)	4,475 (20.2%)	968 (21.1%)	
≥ high school	11,416 (79.6%)	9,276 (79.8%)	2,140 (78.9%)	
BMI category, %				0.257
Obesity	12,414 (74.0%)	10,088 (73.7%)	2,326 (75.2%)	
Overweight	4,216 (24.7%)	3,477 (25.0%)	739 (23.2%)	
Under/normal weight	229 (1.4%)	186 (1.3%)	43 (1.5%)	
Marital status, %				0.003
Not married	6,120 (34.1%)	4,978 (33.3%)	1,142 (37.5%)	
Married/living with partner	10,317 (64.0%)	8,431 (64.8%)	1,886 (60.5%)	
Missing	422 (1.9%)	342 (1.9%)	80 (1.9%)	
Household income, %				0.062
Low	5,130 (22.1%)	4,201 (22.4%)	929 (20.6%)	
Moderate	6,023 (34.3%)	4,859 (33.8%)	1,164 (37.0%)	
High	4,328 (36.9%)	3,554 (37.3%)	774 (35.4%)	
Missing	1,378 (6.6%)	1,137 (6.5%)	241 (7.0%)	
Hypertension, %				<0.001
Yes	10,546 (61.3%)	8,569 (60.3%)	1,977 (65.6%)	
No	6,313 (38.7%)	5,182 (39.7%)	1,131 (34.4%)	
Diabetes, %				<0.001
Yes	4,265 (20.8%)	3,359 (19.4%)	906 (27.1%)	
No	12,594 (79.2%)	10,392 (80.6%)	2,202 (72.9%)	
Smoking status, %				<0.001
Current	2,982 (18.7%)	2,450 (19.0%)	532 (17.6%)	
Former	4,425 (26.8%)	3,464 (25.3%)	961 (33.6%)	
Never	8,983 (53.3%)	7,452 (54.5%)	1,531 (47.8%)	
Missing	469 (1.1%)	385 (1.1%)	84 (1.0%)	

1 Continuous variables are presented as median and interquartile range (Q1, Q3); categorical variables are presented as unweighted numbers and weighted percentages.

2 Wilcoxon Rank-Sum test for continuous variables; Chi-Squared test with Rao & Scott’s second-order correction for categorical variables.

### Associations of NLR and ACM in MASLD

**Figure 3:**
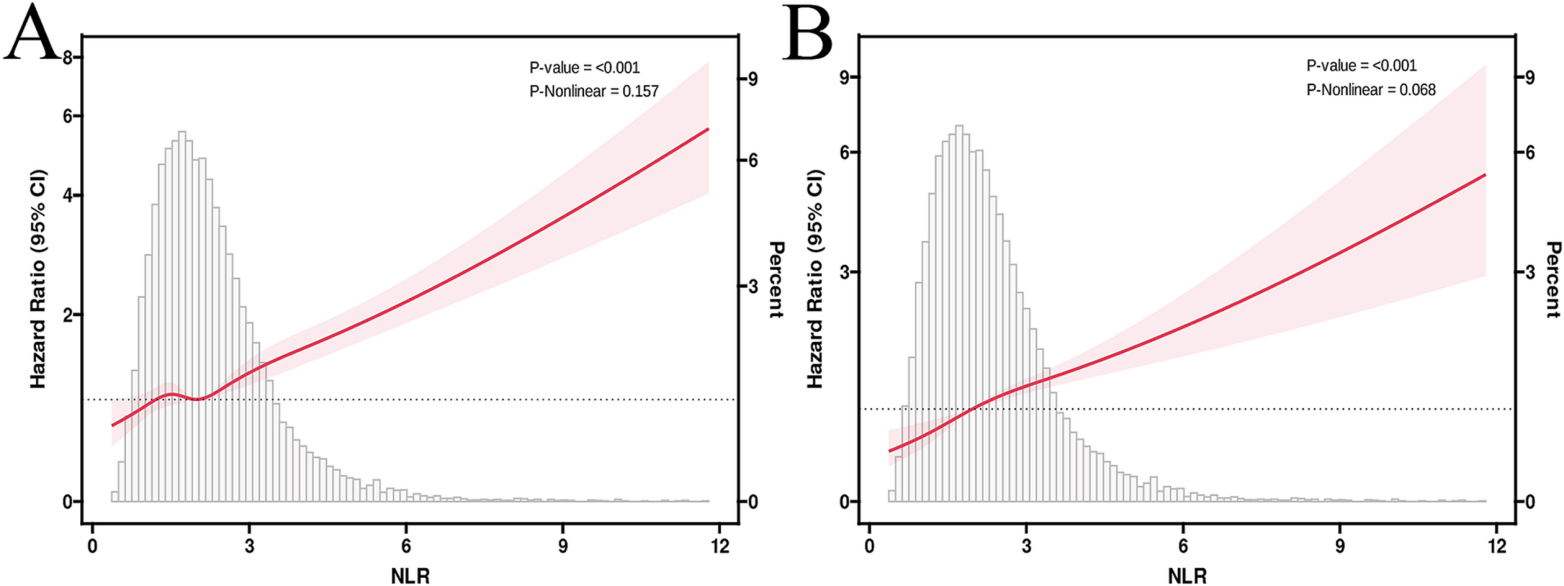
RCS curves illustrate the associations between NLR, ACM **(A)**, and CVM **(B)**. HRs were adjusted for age, gender, race/ethnicity, marital status, education level, household income, energy intake, BMI, smoking status, diabetes, hypertension, TG, WC, GGT, HDL,ALT and FIB-4 index.

The multivariable-adjusted RCS plot demonstrated a positive linear correlation between NLR and ACM in MASLD participants (*p* for non-linear = 0.157; Figure 3A). When analyzing the NLR as a continuous variable, a one-unit elevation in NLR was linked to a hazard ratio (HR) of 1.23 (95% confidence interval [CI]: 1.19–1.27, *p* < 0.001) for ACM in unadjusted Model 1. Following multivariable adjustment, a one-unit elevation in NLR corresponded to a raised risk of ACM by 17% (HR 1.17, 95% CI: 1.12–1.22, *p* < 0.001, Model 2) and 16% (HR 1.16, 95% CI: 1.12–1.21, *p* < 0.001, Model 3). The higher NLR group, considering the NLR as a categorical variable, exhibited an HR for ACM of 1.87 (95% CI: 1.67–2.11, *p* < 0.001, Model 1), an HR of 1.42 (95% CI: 1.27–1.60, *p* < 0.001, Model 2), and an HR of 1.38 (95% CI: 1.23–1.55, *p* < 0.001, Model 3) in comparison to the lower reference group (Table 2).

**Table 2:** Associations of NLR with ACM and CVM in MASLD participants.

Variable	Model 1		Model 2		Model 3	
	HR (95% CI)	*p*	HR (95% CI)	*p*	HR (95% CI)	*p*
ACM						
NLR	1.23 (1.19, 1.27)	<0.001	1.17 (1.12, 1.22)	<0.001	1.16 (1.12, 1.21)	<0.001
NLR group						
Lower NLR	Ref		Ref		Ref	
Higher NLR	1.87 (1.67, 2.11)	<0.001	1.42 (1.27, 1.60)	<0.001	1.38 (1.23, 1.55)	<0.001
CVM						
NLR	1.27 (1.22, 1.32)	<0.001	1.21 (1.14, 1.28)	<0.001	1.21 (1.15, 1.27)	<0.001
NLR group						
Lower NLR	Ref		Ref		Ref	
Higher NLR	2.30 (1.90, 2.79)	<0.001	1.66 (1.35, 2.04)	<0.001	1.64 (1.32, 2.03)	<0.001

Model 1: Unadjusted; Model 2: Adjusted for age, gender, race/ethnicity, marital status, and education level; Model 3: Further adjusted for household income, energy intake, BMI, smoking status, diabetes, hypertension, TG, WC, GGT, HDL, ALT and FIB-4 index.

### Correlations of NLR and CVM in MASLD

The multivariable-adjusted RCS plot demonstrated a positive linear link between NLR and CVM in MASLD, with no significant *p*-value for nonlinearity (*p* = 0.068; Figure 3B). Analyzing NLR as a continuous variable revealed that each one-unit elevation in NLR corresponded to an HR of 1.27 (95% CI: 1.22–1.32, *p* < 0.001) for CVM in unadjusted Model 1. Following multivariable adjustment, a one-unit elevation in NLR was related to a 21% rise in the CVM risk (HR 1.21, 95% CI: 1.14–1.28, *p* < 0.001, Model 2) and a 21% rise in the CVM risk (HR 1.21, 95% CI: 1.15–1.27, *p* < 0.001, Model 3). The higher NLR group, considering the NLR as a categorical variable, exhibited an HR for CVM of 2.30 (95% CI: 1.90–2.79, *p* < 0.001, Model 1), 1.66 (95% CI: 1.35–2.04, *p* < 0.001, Model 2), and 1.64 (95% CI: 1.32–2.03, *p* < 0.001, Model 3) compared to the lower reference group (Table 2).

### Kaplan–Meier survival curve analyses

**Figure 4:**
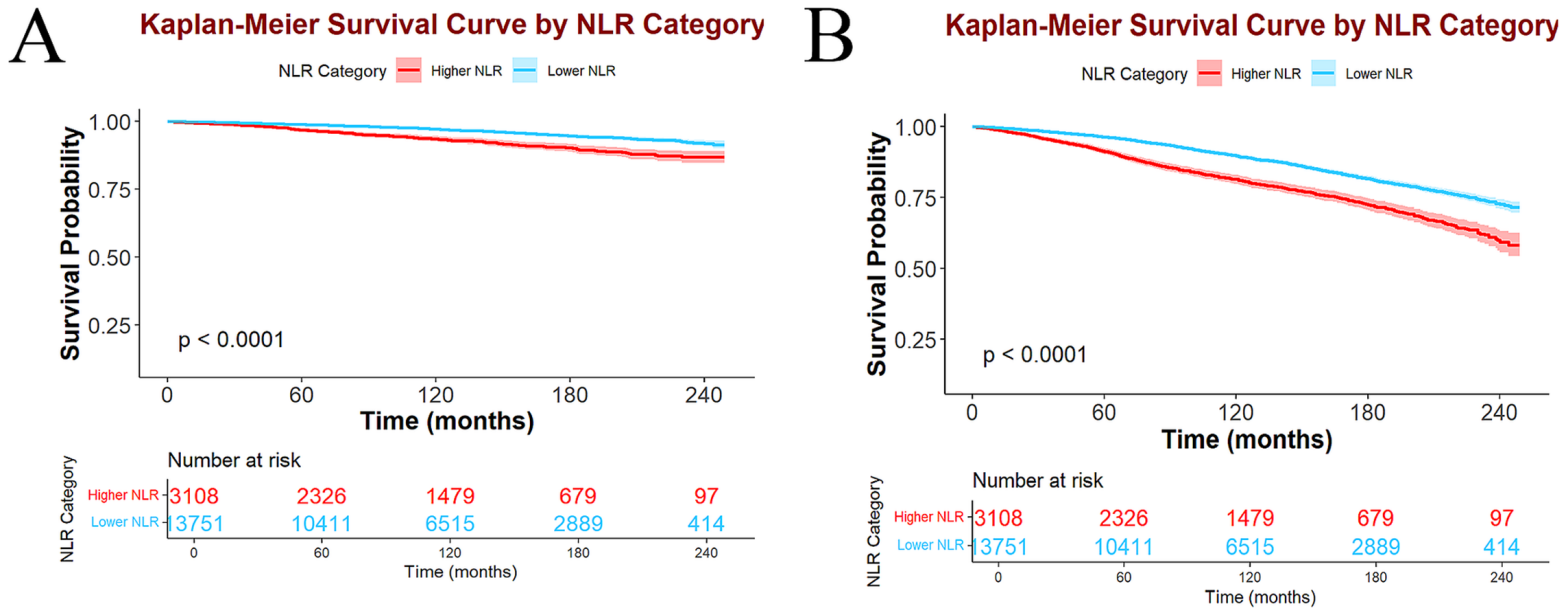
Kaplan–Meier survival curves and participant number at risk in higher and lower NLR groups of ACM **(A)** and CVM **(B)**.

Kaplan–Meier survival curves manifested a statistically significant variance in survival between the higher and lower NLR groups. Specifically, the higher NLR group had lower survival probabilities for both ACM (Figure 4A) and CVM (Figure 4B) than the lower NLR group, with all *p* < 0.0001.

### Subgroup and sensitive analyses

In subgroup analyses classified by age, gender, BMI, hypertension, and diabetes, elevated NLR was consistently correlated with an elevated risk of ACM and CVM across all subgroups. Notably, no significant interaction between these variables and NLR was observed (Table 3). Sensitivity analyses were performed restricted to individuals interviewed between 2003 and 2008, with subsequent recalibration of the weights. Consistent associations between NLR and mortality were observed (Table 4).

**Table 3:** Subgroup analyses of NLR and mortality risk in MASLD.

Subgroup	ACM			CVM		
	HR (95% CI)	*p*-value	P for interaction	HR (95% CI)	*p*-value	P for interaction
Age (years)			0.475			0.267
< 65	1.17 (1.10, 1.24)	<0.001		1.22 (1.12, 1.32)	<0.001	
≥ 65	1.15 (1.10, 1.21)	<0.001		1.20 (1.10, 1.31)	<0.001	
Gender			0.987			0.451
Male	1.15 (1.05, 1.22)	<0.001		1.18 (1.10, 1.26)	<0.001	
Female	1.19 (1.11, 1.27)	<0.001		1.30 (1.15, 1.46)	<0.001	
BMI			0.064			0.062
Obesity	1.18 (1.12, 1.23)	<0.001		1.26 (1.18, 1.35)	<0.001	
Overweight	1.13 (1.05, 1.23)	0.001		1.11 (0.97, 1.27)	0.136	
Under/normal weight	1.32 (0.87, 2.00)	0.186		1.02 (0.54, 2.24)	0.752	
Hypertension			0.291			0.643
Yes	1.19 (1.14, 1.24)	<0.001		1.24 (1.17, 1.31)	<0.001	
No	1.06 (0.97, 1.15)	0.218		1.05 (0.78, 1.24)	0.561	
Diabetes			0.057			0.395
Yes	1.13 (1.07, 1.19)	<0.001		1.15 (1.04, 1.26)	0.006	
No	1.20 (1.13, 1.27)	<0.001		1.28 (1.19, 1.38)	<0.001	

HRs were adjusted for age, gender, race/ethnicity, marital status, education level, household income, energy intake, BMI, smoking status, diabetes, hypertension, TG, WC, GGT, HDL, ALT and FIB-4 index, excluding the stratifying factors.

**Table 4:** Sensitivity analyses of NLR and mortality risk in MASLD.

Variable	Model 1		Model 2		Model 2	
	HR (95% CI)	*p*	HR (95% CI)	*p*	HR (95% CI)	*p*
ACM						
NLR	1.25 (1.18, 1.32)	<0.001	1.16 (1.08, 1.25)	<0.001	1.15 (1.07, 1.24)	<0.001
NLR group						
Lower NLR	Ref		Ref		Ref	
Higher NLR	1.81 (1.54, 2.12)	<0.001	1.37 (1.14,1.65)	<0.001	1.35 (1.13, 1.62)	<0.001
CVM						
NLR	1.40 (1.26, 1.54)	<0.001	1.30 (1.15, 1.46)	<0.001	1.29 (1.14, 1.46)	<0.001
NLR group						
Lower NLR	Ref		Ref		Ref	
Higher NLR	2.52 (1.91, 3.32)	<0.001	1.86 (1.37, 2.53)	<0.001	1.90 (1.39, 2.59)	<0.001

Model 1: Unadjusted; Model 2: Adjusted for age, gender, race/ethnicity, marital status, and education level; Model 3: Further adjusted for household income, energy intake, BMI, smoking status, diabetes, hypertension, TG, WC, GGT, HDL,ALT and FIB-4 index.

### Predictive value of NLR for ACM and CVM in MASLD

**Figure 5:**
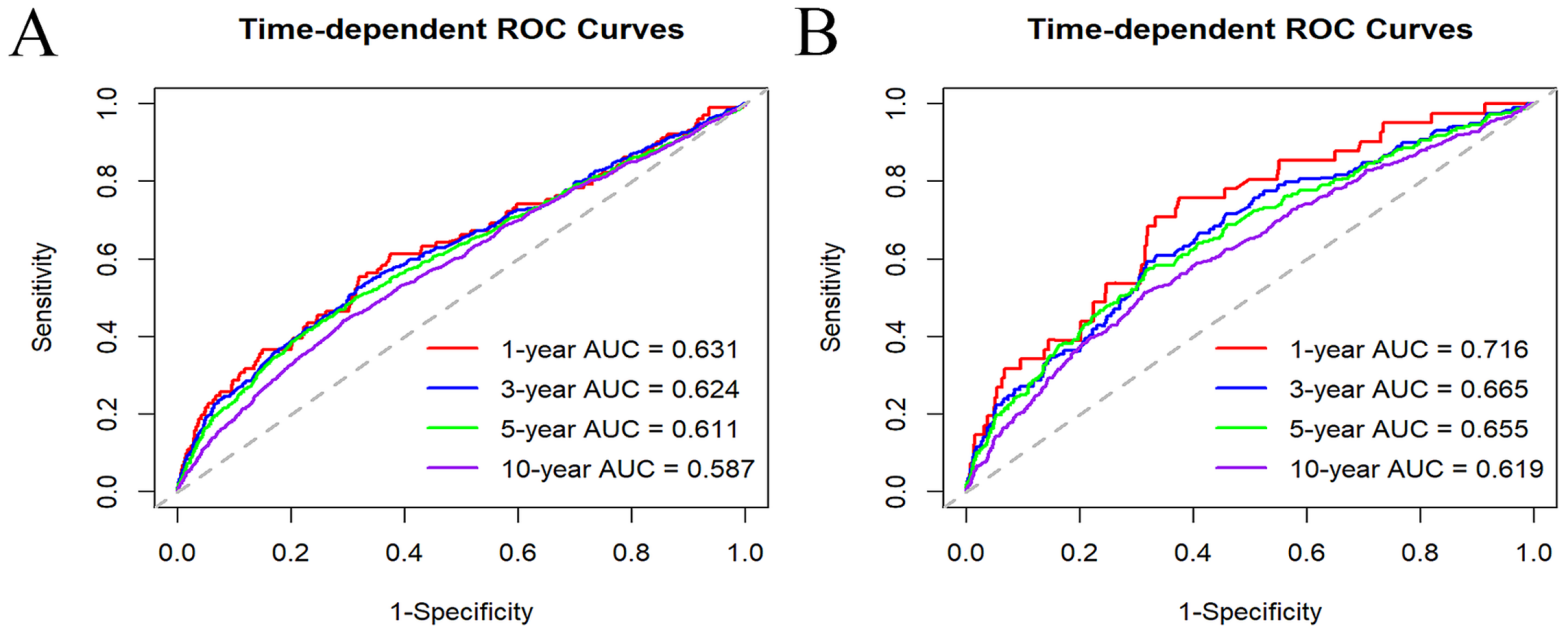
Time-dependent ROC curves of NLR in predicting 1, 3, 5, and 10 years of ACM **(A)** and CVM **(B)**.

Time-dependent ROC analyses were deployed to detect the prognostic utility of NLR for predicting ACM and CVM in MASLD. The AUC values for NLR predicting ACM were 0.631, 0.624, 0.611, and 0.587 for 1-, 3-, 5-, and 10-year intervals, respectively. For CVM, the AUC values were 0.716, 0.665, 0.655, and 0.619 for the same respective time intervals. These findings indicate that NLR is a more effective predictor of short-term mortality than long-term mortality and is more predictive of CVM than ACM (Figure 5).

## Discussion

The correlations between NLR and mortality risk in MASLD participants were assessed in this study. Our results elucidated a positive link between elevated NLR and raised mortality, including ACM and CVM, identifying NLR as an independent risk factor for poor survival. Post-adjustment for prevalent covariates, these associations were moderately alleviated. To our knowledge, this is the first study that explores the prognostic significance and dose–response correlations between NLR and mortality risk in the MASLD.

The possible mechanisms correlating the NLR to mortality in MASLD likely involve inflammatory processes and immune responses (13). Neutrophils, the immune system’s essential components, contribute to the progression of steatosis to steatohepatitis through mechanisms including phagocytosis, cytokine secretion, reactive oxygen species production, and the neutrophil extracellular trap formation (28). Neutrophil infiltration is a defining feature of steatohepatitis, and in patients with this condition, neutrophils exhibit increased activity and functional changes that exacerbate liver inflammation and ultimately lead to fibrosis (29). In animal models, neutrophil depletion has ameliorated high-fat diet-provoked SLD by reducing liver lipid accumulation and the inflammatory response (30). Different types of lymphocytes play both protective and deleterious roles in SLD. B cells, the predominant lymphocytes in the liver, produce antibodies and cytokines that can either exacerbate or alleviate liver injury depending on the microenvironment (31). T cells, including subsets such as Th1/2/17 and Treg cells, also have differential effects on SLD. For example, cytotoxic T cells and Th17 generally enhance liver inflammation and fibrosis, whereas Treg cells tend to have a protective effect by mitigating inflammation (32).

The associations between NLR and NAFLD have been explored, yielding inconsistent findings. A cohort study involving 101 patients elucidated a significant positive link between NLR and the activity score of NAFLD, as well as its components, including steatosis, inflammation, and ballooning (13). Conversely, a cross-sectional study of 231 biopsy-confirmed NAFLD patients in China found negative relations between elevated NLR levels and advanced inflammatory activity and significant fibrosis in NAFLD patients (33). In contrast to previous studies with smaller sample sizes, we utilized a large, nationally representative cohort and confirmed that elevated NLR was positively correlated with poor prognosis in MASLD. Importantly, this correlation remained unaffected by gender, age, BMI, hypertension, and diabetes. Our findings indicate that NLR may act as a prognostic biomarker to aid in the clinical management of MASLD. Identifying novel prognostic biomarkers could help healthcare providers develop personalized treatment strategies for this population. However, the NLR predictive power is relatively limited (AUC < 0.7, except for 1-year CVM), underscoring its limitations as a sole predictor. Consequently, it is suitable to integrate NLR with other biomarkers in clinical practice to enhance predictive accuracy.

In addition, there is no consensus on normal NLR values, although a range of 1–2 is commonly suggested as normal, 2–3 as a gray region elucidating subclinical inflammation, and > 3 as indicative of inflammation (34). Our study aligned with these recommendations and identified an optimal NLR cut-off value of 2.88, which was related to the most significant survival outcomes in MASLD. Tailoring specific NLR cut-off values to different patient groups may enhance this marker’s predictive accuracy in clinical practice.

The study possesses several notable strengths. First, it includes a large sample of participants and has a long follow-up period, which ensures robust conclusions and adequate statistical power. Second, the exclusive inclusion of NHANES participants reduced the risk of selection bias. Third, we utilized multiple statistical techniques to determine the correlations between NLR and mortality risk. Additionally, we controlled numerous covariates to determine the independent associations of NLR with mortality outcomes in MASLD.

However, there are many limitations to this study. The diagnosis of MASLD was established using a FLI score of ≥60, rather than relying on histological or ultrasonographic assessments. While the FLI score offers the benefit of simplicity in identifying patients with MASLD, it is not regarded as the gold standard. Consequently, this reliance on the FLI score may lead to misclassification, resulting in either an underestimation or overestimation of the prevalence of MASLD, which may, in turn, influence the associations observed in our analysis. Additionally, despite controlling for multiple demographic and socioeconomic factors, some residual confounders, such as participants’ medication use, were not accounted for. Moreover, the NLR calculation was based on baseline data, limiting our ability to assess longitudinal changes in this biomarker and its impact on clinical outcomes in MASLD over time. Furthermore, since the United States population provided the findings, it is crucial to evaluate the generalizability of these findings to other regions with diverse racial demographics. Accurate validation of these results requires further well-designed longitudinal and prospective studies.

## Conclusion

Our study illustrates a positive link between higher NLR and a raised risk of both CVM and ACM in MASLD adults. Consequently, NLR may be an easily and simply calculable clinical biomarker for MASLD management.

## Data Availability

The raw data supporting the conclusions of this article will be made available by the authors, without undue reservation.

## Footnotes

^1^ https://www.cdc.gov/nchs/nhanes/

^2^ https://www.cdc.gov/nchs/datalinkage/mortality-public.htm
